# Supplementary material for: Unequal airborne exposure to toxic metals associated with race, ethnicity, and segregation in the USA
Source: Nat Commun. 2022 Nov 1;13:6329. doi: 10.1038/s41467-022-33372-z (PMC9626599; doi:10.1038/s41467-022-33372-z)
Supplement: Supplementary file 1 — Supplementary Info [file 41467_2022_33372_MOESM1_ESM.pdf]

Supplementary Information for

Unequal airborne exposure to toxic metals associated with race, ethnicity, and segregation in the USA

John K. Kodros, Michelle L. Bell, Francesca Dominici, Christian L'Orange, Krystal J. Godri Pollitt, Scott Weichenthal, Xiao Wu, John Volckens

John K. Kodros
Email: jkkodros.research@gmail.com

Table S1. Summary statistics for the year 2019 for sites that meet the inclusion criteria.

Species	Mean	SD	5th	25th	50th	75th	95th	% below MDL	Moran's I
$PM_{2.5}$ [µg m^{-3}]	5.6	2.6	1.9	2.9	6.1	7.9	9.4	-	0.46
Cu [ng m^{-3}]	2.3	3.2	0.1	0.3	1.0	3.3	6.5	63	0.10
Zn [ng m^{-3}]	8.0	10	0.8	1.7	6.3	10	22	14	0.26
Ni [ng m^{-3}]	0.5	0.5	0.03	0.1	0.5	0.8	1.1	75	0.32
Cr [ng m^{-3}]	1.2	1.4	0.04	0.1	1.1	2.0	3.1	67	0.31
Pb [ng m^{-3}]	1.8	1.7	0.1	0.4	1.6	2.6	4.8	78	0.38
V [ng m^{-3}]	0.2	0.2	0.04	0.1	0.1	0.2	0.4	75	0.36
Fe [ng m^{-3}]	56	66	10	22	44	67	135	7	0.14
Mn [ng m^{-3}]	1.5	2.1	0.3	0.6	1.1	1.7	3.3	56	0.20
Ti [ng m^{-3}]	3.0	1.9	0.9	1.5	2.6	4.1	6.9	35	0.40

Table S2. Percent increase in concentration corresponding to a 10% increase in dissimilarity index modeled through univariate regression.

	All Sites		Urban Sites	
Species	Coefficient [%]	95th CI [%]	Coefficient [%]	95th CI [%]
$PM_{2.5}$	5.0	2.9-7.1	2.2	0.1-4.3
Cu	11	6.1-15	10	2.6-19
Zn	9.4	5.8-13	11	6.1-17
Ni	13	8.7-18	8.6	4.9-12
Cr	16	10-22	9.6	4.4-15
Pb	9.4	5.4-13	5.2	1.2-9.4
V	3.6	1.4-5.9	4.0	-0.5-8.6
Fe	6.6	4.1-9.3	7.8	2.8-13
Mn	5.7	3.1-8.4	9.4	2.7-17
Ti	3.1	0.9-5.3	-0.8	-5.6-4.3

Table S3. Percent increase in concentration corresponding to a 10% increase in dissimilarity index controlling for regional factors in a fixed effects model. Values in the parenthesis represent the 95th percentile confidence interval.

Species	Coefficient	Border	Midwestern	Mountains	Northeastern	Southern	Western
$PM_{2.5}$	4.0 (1.9-6.3)	16 (14-19)	24 (21-26)	18 (15-21)	20 (18-23)	23 (20-26)	17 (14-19)
Cu	8.9 (4.2-14)	2.7 (-3.2-9.0)	8.7 (3.9-13.7)	-0.3 (-5.4-5.0)	9.6 (4.8-15)	11 (5.3-17)	1.7 (-3.5-7.2)
Zn	6.8 (3.3-10.3)	13 (7.7-18)	29 (25-33)	15 (11-19)	26 (22-31)	24 (19-29)	14 (10-19)
Ni	12 (7-17)	-11 (-16 - -6)	-1 (-5.2-3.6)	-11 (-15 - -5.8)	-1.4 (-5.6-3.0)	-1.1 (-6.0-4.1)	-12 (-16 - -6.9)
Cr	13 (7.6-20)	-7.6 (-14 - -1)	8.0 (2.4-14)	-3.6 (-9.4-2.6)	6.6 (1.2-12)	6.3 (-0.1-13)	-6.1 (-12 - -0)
Pb	6.5 (2.8-10)	0.2 (-4.4-5.1)	13 (8.6-17)	-0.9 (-5.0-3.3)	9.2 (5.4-13)	9.2 (4.8-14)	-2.5 (-6.5-1.8)
V	3.6 (1.5-5.9)	-14 (-16 - - 11)	-15 (-16 - - 12)	-18 (-20 - -16)	-17 (-19 - -15)	-12 (-14 - -9.4)	-18 (-20 - -16)
Fe	6.7 (3,9-9.6)	51 (45-56)	50 (46-55)	48 (44-53)	45 (42-50)	52 (47-57)	45 (41-50)
Mn	5.3 (2.5-8.2)	4.7 (1.0-8.6)	7.5 (4.6-10)	3.5 (0.3-6.8)	2.5 (-0.3-5.3)	6.0 (2.7-9.4)	1.6 (-1.6-4.9)
Ti	4.2 (1.99-6.4)	17 (14-21)	8.4 (6.1-11)	13 (11-16)	8.7 (6.4-11)	16 (14-19)	12 (9.0-15)

Table S4. Univariate linear regression model results showing the percent increase (and 95th CI) in concentration corresponding to a 10% increase in dissimilarity index for the NHB, hispanic, asian, and native populations relative to the NHW population.

Species	NHB	Hispanic	Asian	Native American
$PM_{2.5}$	5.0 (2.9-7.1)	3.9 (2.7-5.0)	1.4 (0.3-2.4)	2.7 (1.6-3.8)
Cu	11 (6.1-15)	9.7 (7.1-12)	3.4 (0.8-6.1)	7.6 (5.0-10)
Zn	9.4 (5.8-13)	8.2 (6.1-10)	2.9 (0.8-5.1)	5.9 (3.8-8.1)
Ni	13 (9.7-18)	11 (8.5-14)	3.8 (1.0-6.6)	8.6 (5.9-11)
Cr	16 (10-22)	13 (9.8-16)	4.4 (1.1-7.8)	9.9 (6.6-13)
Pb	9.4 (5.4-13)	8.5 (6.3-11)	3.4 (1.1-5.8)	6.8 (4.5-9.1)
V	3.6 (1.4-5.9)	3.1 (1.8-4.4)	1.3 (0.0-2.7)	2.5 (1.2-3.9)
Fe	6.6 (4.1-9.3)	5.3 (3.8-6.8)	2.0 (0.5-3.6)	4.2 (2.6-5.7)
Mn	5.7 (3.1-8.4)	4.8 (3.3-6.4)	2.0 (0.4-3.5)	3.6 (2.1-5.2)
Ti	3.1 (0.9-5.3)	3.3 (2.0-4.6)	1.5 (0.3-2.8)	2.7 (1.6-3.8)

Table S5. Multivariable linear regression model showing the percent increase (and 95th CI) in concentration corresponding to a 10% increase in dissimilarity index ("DI coefficient") and 10% increase in the non-Hispanic Black population ("NHB coefficient").

Species	DI Coefficient	NHB Coefficient
$PM_{2.5}$	1.4 (-0.2-3.0)	2.1 (1.7-2.4)
Cu	4.1 (0.8-7.5)	5.2 (4.5-6.0)
Zn	4.3 (1.6-7.2)	4.1 (3.4-4.7)
Ni	6.5 (3.1-10.0)	5.4 (4.7-6.2)
Cr	7.8 (3.5-12)	6.3 (5.3-7.2)
Pb	3.9 (0.9-6.9)	4.4 (3.7-5.1)
V	1.2 (-0.8-3.2)	1.9 (1.4-2.4)
Fe	3.7 (1.5-5.9)	2.4 (1.9-2.9)
Mn	3.0 (0.7-5.3)	2.2 (1.7-2.8)
Ti	1.4 (-0.7-3.5)	1.4 (0.9-1.9)

Table S6. Multivariable linear regression model showing the percent increase (and 95th CI) in concentration corresponding to a 10% increase in dissimilarity index ("DI coefficient") and 10% increase in the non-Hispanic White population ("NHW coefficient").

Species	DI Coefficient	NHW Coefficient
$PM_{2.5}$	4.4 (2.3-6.6)	-1.4 (-2.9-0.2)
Cu	8.7 (4.2-13)	-6.3 (-9.7 - -2.9)
Zn	9.0 (5.3-13)	-1.3 (-4.2-1.8)
Ni	12 (7.1-17)	-5.2 (-8.7 - -1.6)
Cr	15 (8.9-21)	-3.8 (-8.1-0.7)
Pb	9.0 (4.9-13)	-1.3 (-4.5-2.0)
V	2.4 (0.3-4.6)	-4.2 (-6.0 - -2.4)
Fe	5.2 (2.7-7.8)	-4.9 (-6.9 - -2.9)
Mn	4.9 (2.3-7.6)	-2.9 (-5.0 - -0.7)
Ti	1.5 (-0.5-3.5)	-5.8 (-7.5 - -4.2)

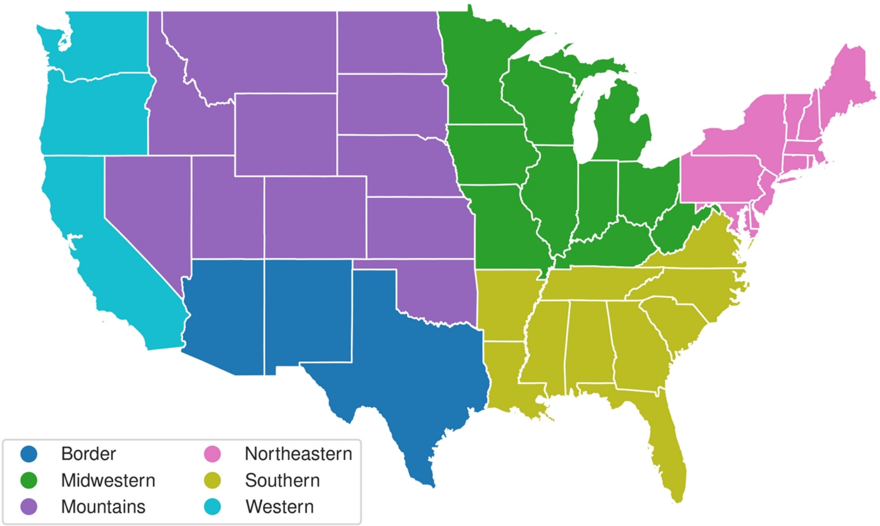

Figure S1. Geographical grouping of states following the definitions in Morello-Frosch and Jesdale (2006).

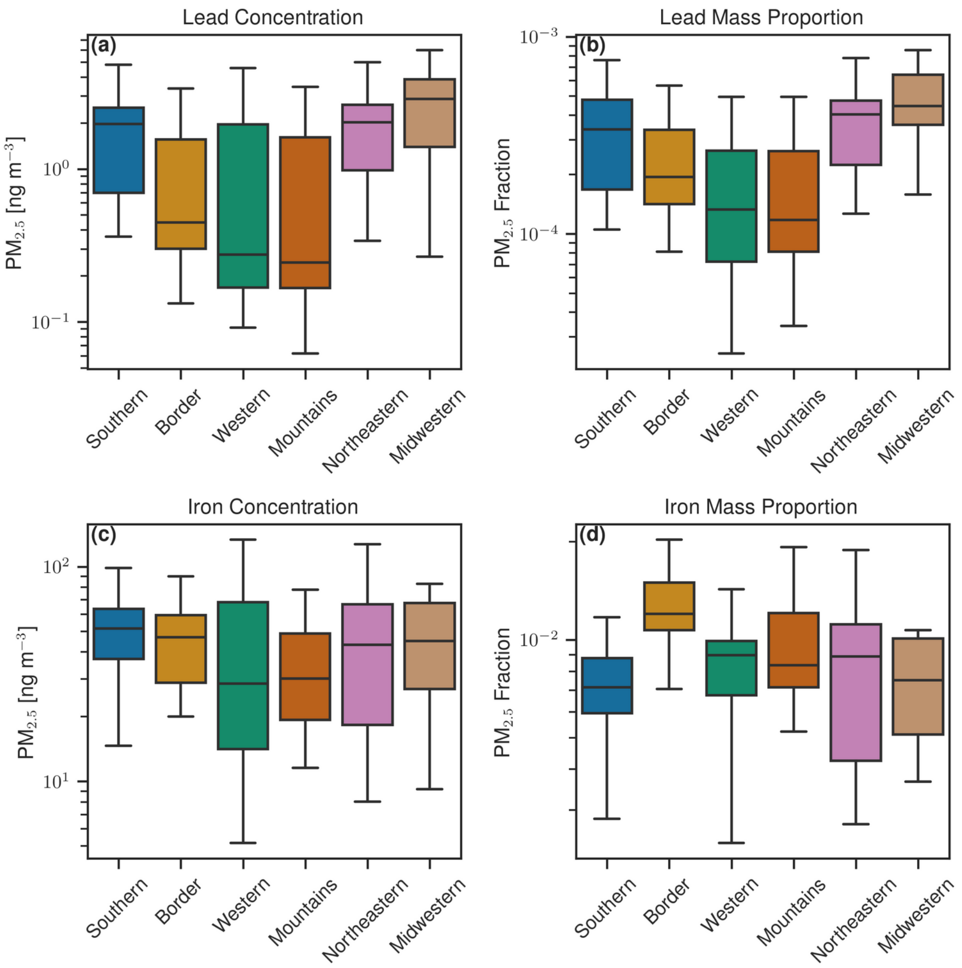

Figure S2. Distribution of annual mean **(a)** lead concentration, **(b)** lead mass proportion, **(c)** iron concentration, and **(d)** iron mass proportion across the six geographic regions shown in Figure S1: Southern (n=38), Border (n=23), Western (n=35), Mountains (n=56), Northeastern (n=41), Midwestern (n=42) states. The box represents the interquartile range, the centerline shows the median, and the whiskers are 1.5 times the interquartile range. Outliers have been omitted for clarity.

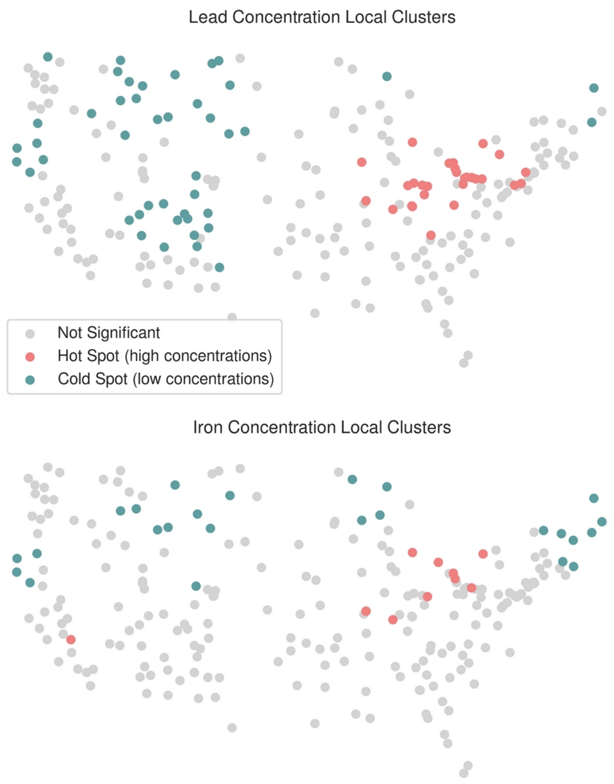

Figure S3. Statistically significant (top) lead and (bottom) iron clusters of hot spots (high concentrations) and cold spots (low concentrations) identified through Local Moran's I.

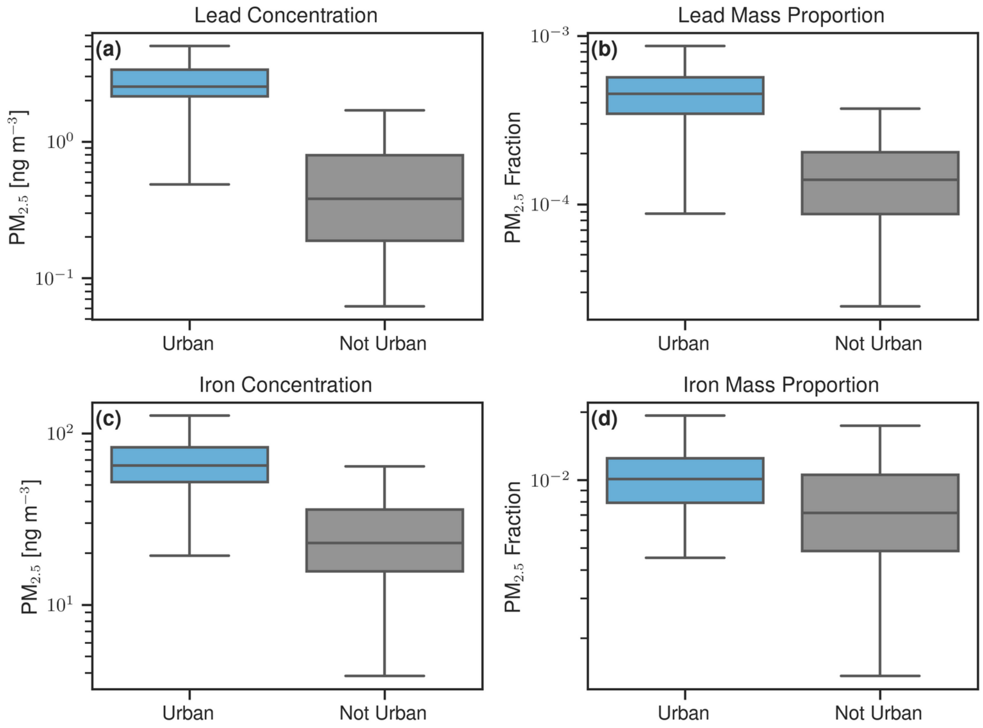

Figure S4. Distribution of annual concentrations in urban areas (n=100) compared to non-urban (n=146) areas for concentrations and mass proportions of (**a, b**) lead and (**c**, **d**) iron. The box represents the interquartile range, the centerline shows the median, and the whiskers are 1.5 times the interquartile range. Outliers have been omitted for clarity.

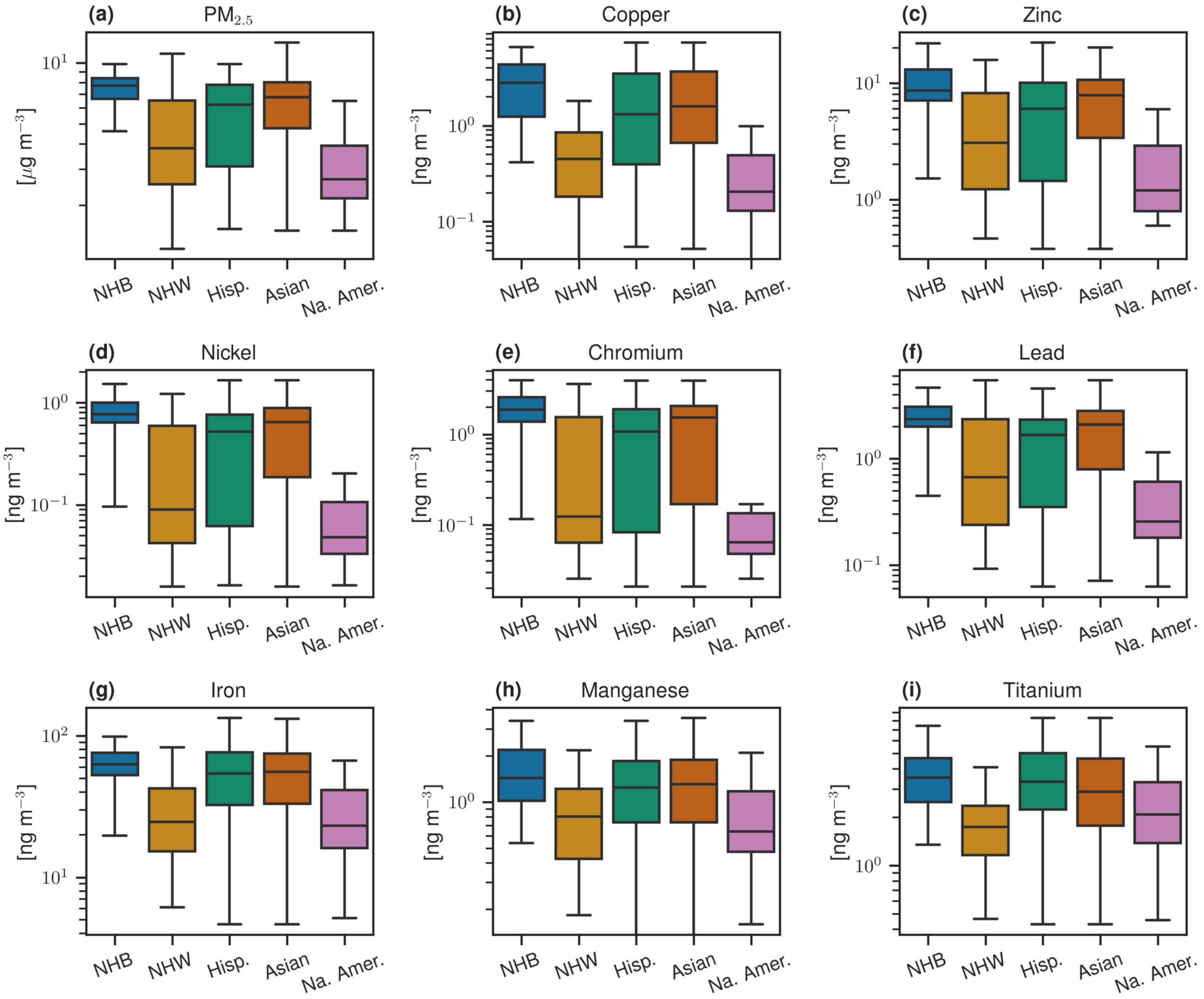

Figure S5. Distribution of **(a)** $PM_{2.5}$, **(b)** copper, **(c)** zinc, **(d)** nickel, **(e)** chromium, **(f)** lead, **(g)** iron, **(h)** manganese, and **(i)** titanium concentrations in counties with above average proportion of non-Hispanic Black ("NHB") (n=74), non-Hispanic White ("NHW") (n=108), Hispanic ("Hisp.") (n=84), Asian (n=128), and Native American ("Na. Amer.") (n=53) population. The box represents the interquartile range, the centerline shows the median, and the whiskers are 1.5 times the interquartile range. Outliers have been omitted for clarity.

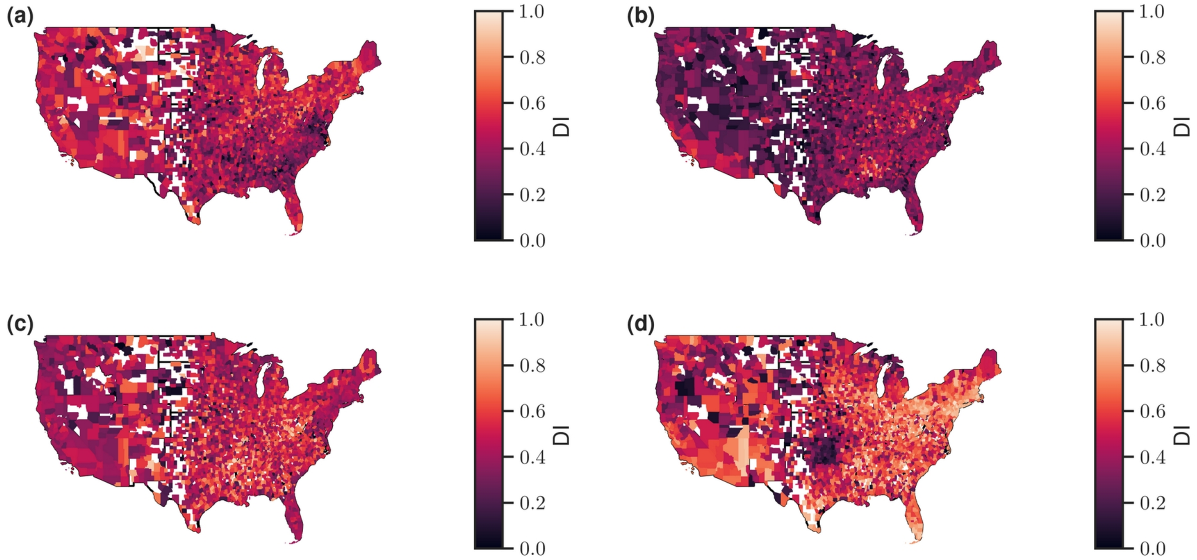

Figure S6. Dissimilarity index by county over the time period 2014-2018 for **(a)** Non-Hispanic Black, **(b)** Hispanic, **(c)** Asian, and **(d)** Native American sub-populations relative to the Non-Hispanic White sub-population.

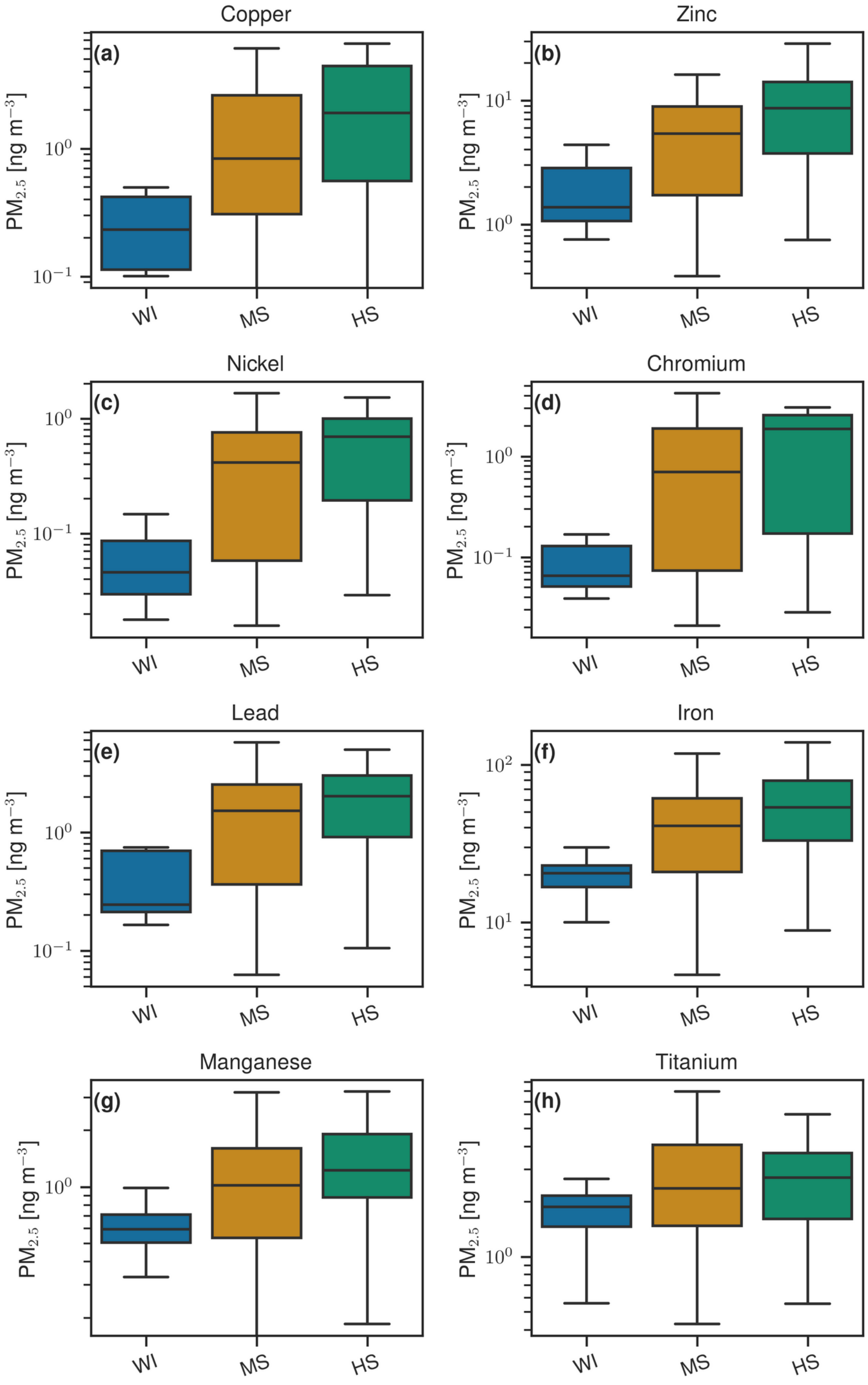

Figure S7. Distribution of concentrations over the NHB-NHW RRS categories for the particulate metals. “WI” stands for well integrated (n=16), “MS” for moderately segregated (n=165), and “HS” for highly segregated (n=47). The box represents the interquartile range, the centerline shows the median, and the whiskers are 1.5 times the interquartile range. Outliers have been omitted for clarity.

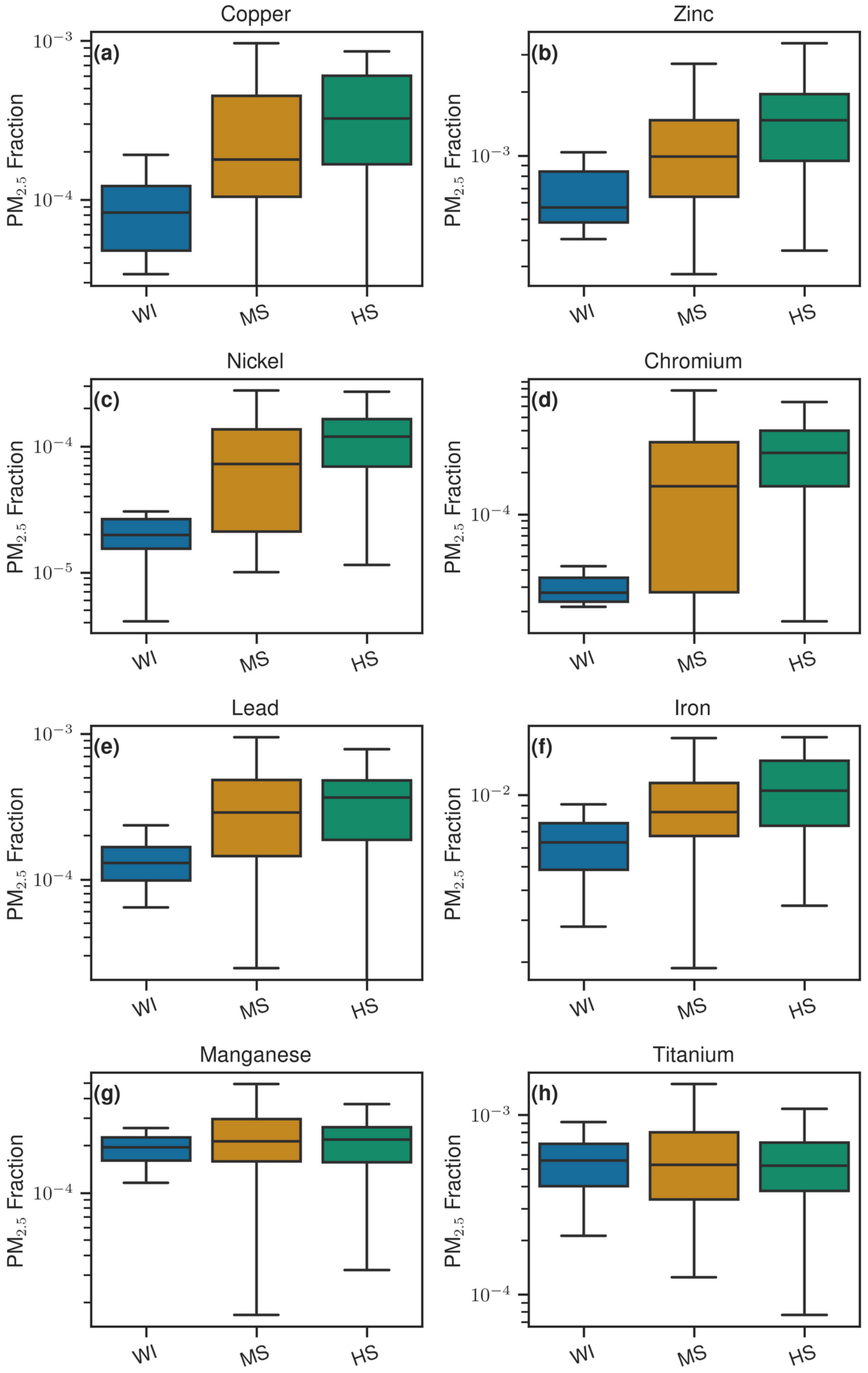

Figure S8. Distribution of trace metal $PM_{2.5}$ mass proportions over NHB-NHW RRS categories. “WI” stands for well integrated (n=16), “MS” for moderately segregated (n=165), and “HS” for highly segregated (n=47). The box represents the interquartile range, the centerline shows the median, and the whiskers are 1.5 times the interquartile range. Outliers have been omitted for clarity.

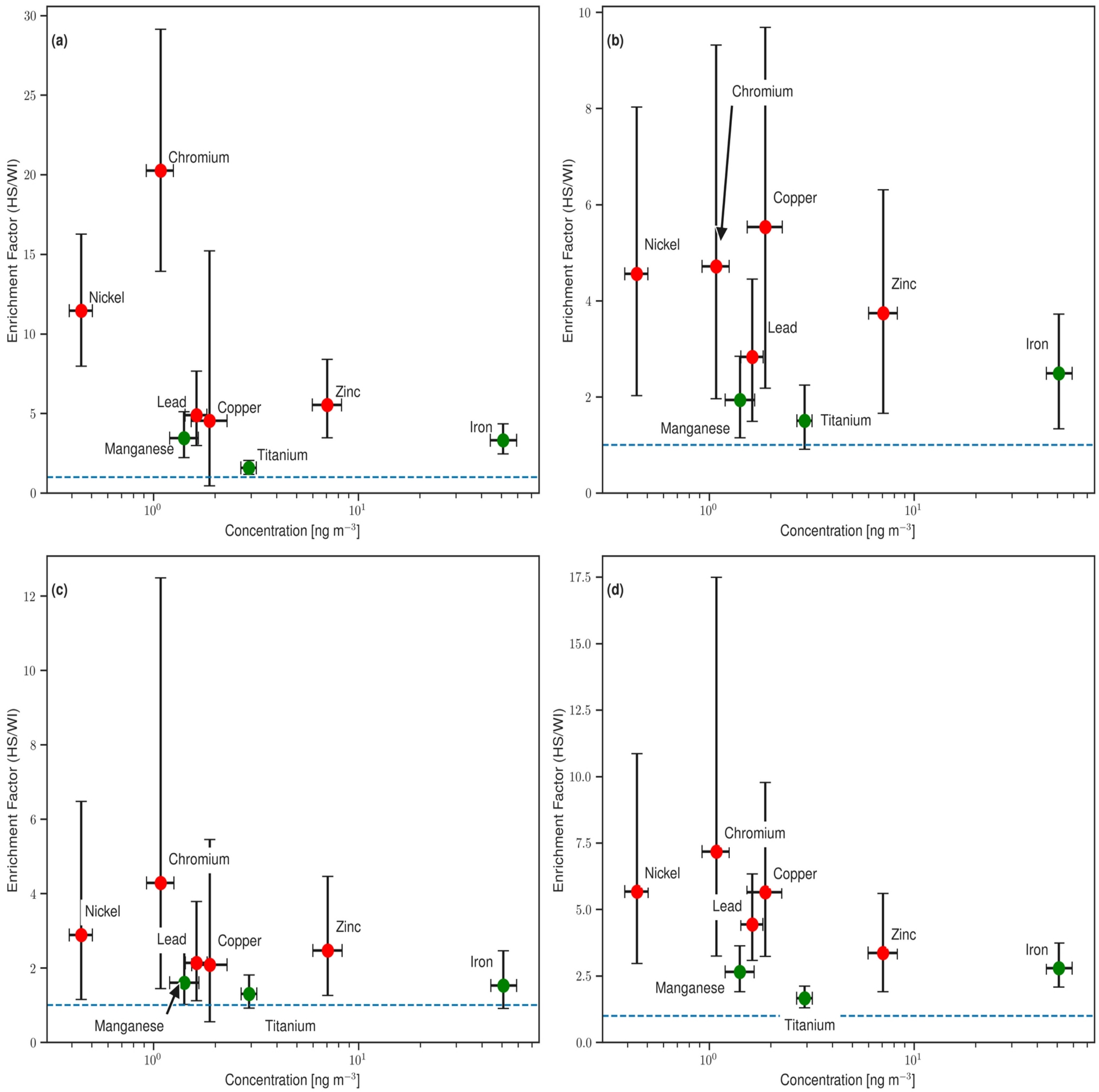

Figure S9. The enrichment factor (the ratio of the mean concentrations) between **a)** NHB-NHW, **b)** Hispanic-NHW, **c)** Asian-NHW, **d)** Native American-NHW highly segregated ("HS") and well-integrated ("WI") counties for the particulate metals as a function of concentration. The x-axis marker denotes the mean concentration (and the error bars depict the 95th CI) in annual mean (2019) concentrations across all sites. The metals commonly associated with anthropogenic emissions are colored red, while the metals associated with natural emissions are colored green. Error bars represent 95th percentile confidence interval. Confidence intervals and center points are calculated through 10,000 random samples with replacement (bootstrap sampling) of size n=47, 7, 24, and 129 for highly segregated counties and n=16, 74, 25, and 24 for well-integrated counties for the NHB-NHW, Hispanic-NHW, Asian-NHW, and Native-American-NHW populations, respectively.

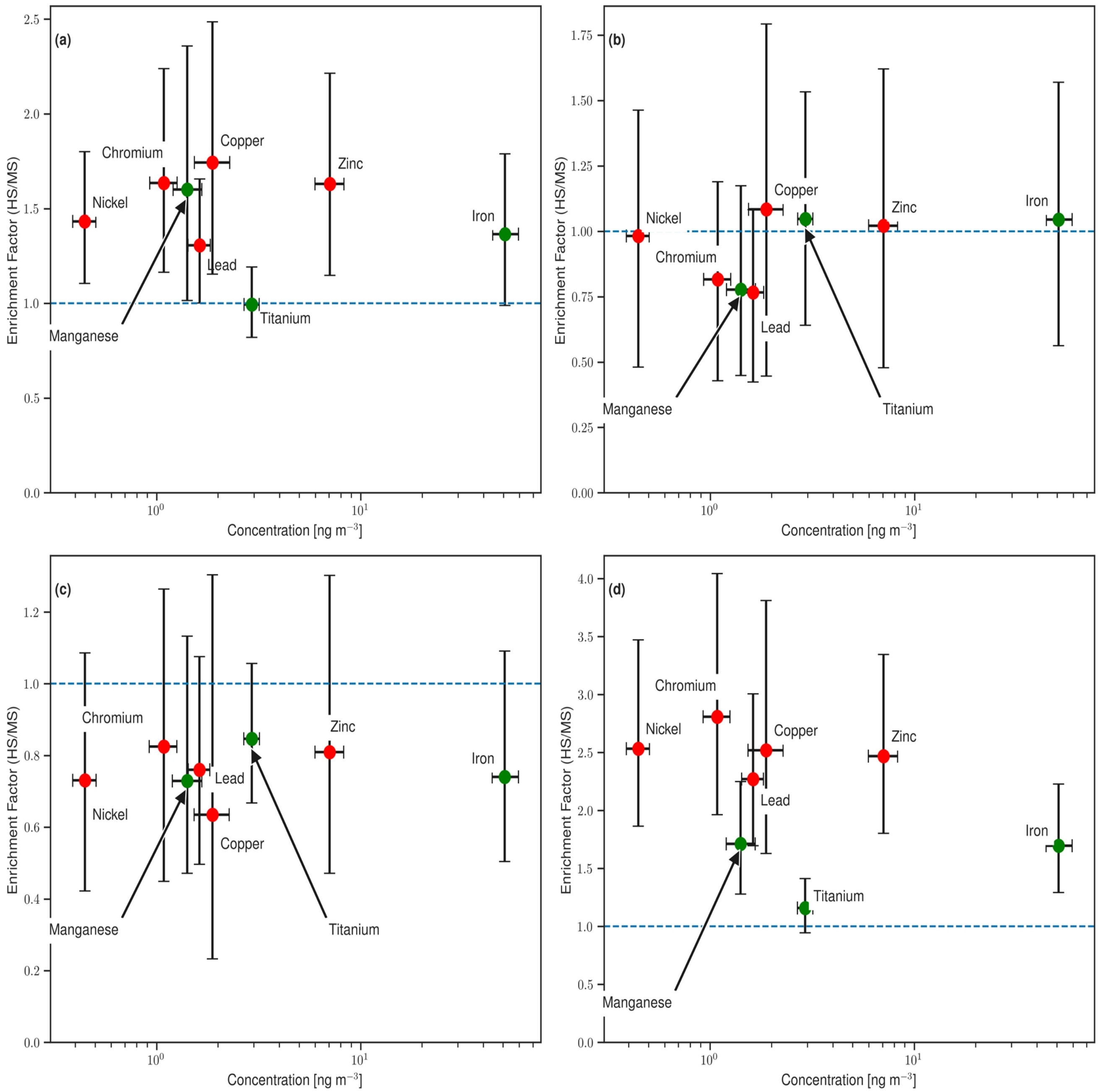

Figure S10. The enrichment factor (the ratio of the mean concentrations) between **a)** NHB-NHW, **b)** Hispanic-NHW, **c)** Asian-NHW, **d)** Native American-NHW highly segregated ("HS") and moderately segregated ("MS") counties for the particulate metals as a function of concentration. The x-axis marker denotes the mean concentration (and the error bars depict the 95th CI) in annual mean (2019) concentrations across all sites. The metals commonly associated with anthropogenic emissions are colored red, while the metals associated with natural emissions are colored green. Error bars represent 95th percentile confidence interval. Confidence intervals and center points are calculated through 10,000 random samples with replacement (bootstrap sampling) of size n=47, 7, 24, and 129 for highly segregated counties and n=165, 149, 180, and 73 for moderately segregated counties for the NHB-NHW, Hispanic-NHW, Asian-NHW, and Native-American-NHW populations, respectively.

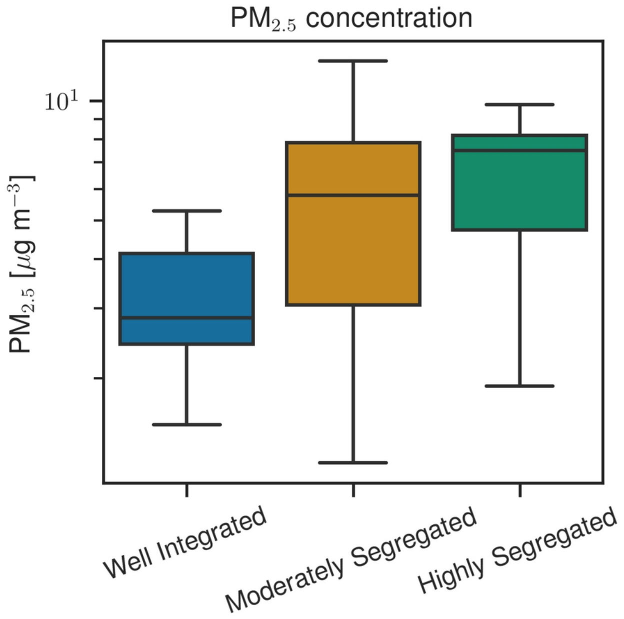

Figure S11. Distributions of total $PM_{2.5}$ concentrations across the RRS categories. The box represents the interquartile range, the centerline shows the median, and the whiskers are 1.5 times the interquartile range. Outliers have been omitted for clarity. RRS categories include: well-integrated (n=16), moderately segregated (n=165), and highly segregated (n=47) US counties for the period 2014-2018.

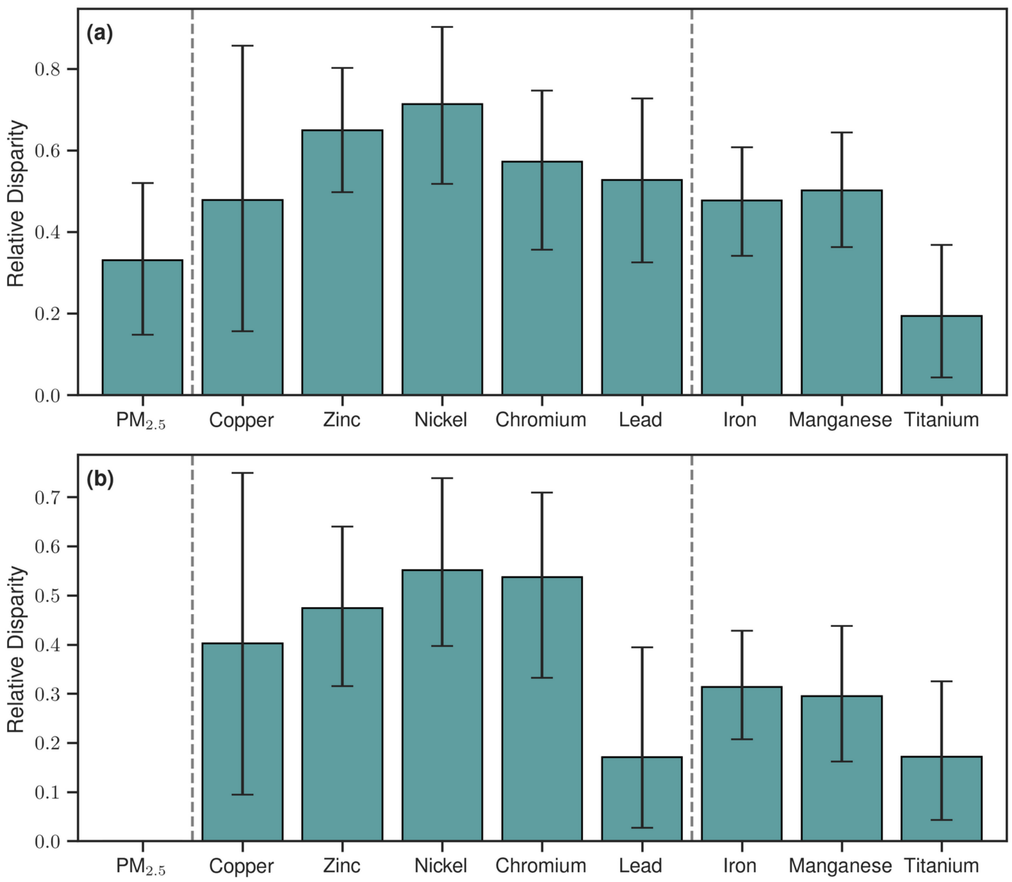

Figure S12. Relative disparities in population-weighted mean **a)** concentration and **b)** $PM_{2.5}$ mass proportion across RRS categories for the time period ranging from 2010-2013. Data is presented with the height of the bars representing the estimate of the mean and the error bars representing the 95th percentile confidence interval. Confidence intervals were estimated using 10,000 random samples with replacement (bootstrap sampling) of size n=58, 157, and 13 for highly segregated, moderately segregated, and well-integrated counties, respectively.

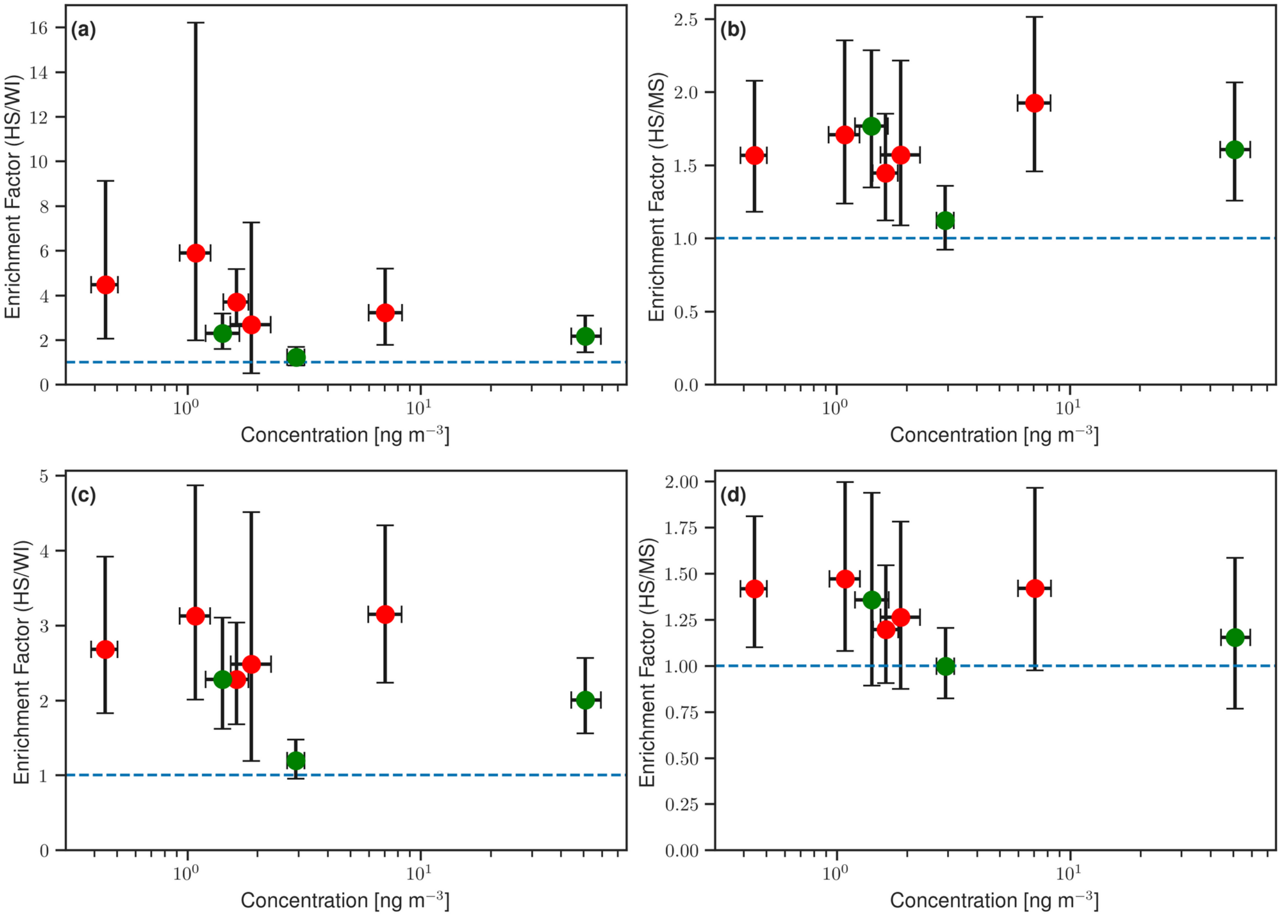

Figure S13. Sensitivity analysis of the enrichment factor (the ratio of the mean) concentration between counties characterized as highly segregated ("HS") to well integrated ("WI") and highly segregated and moderately segregated ("MS") where the categories are determined by an equal percentile grouping of (**a, b)** all US counties and (**c, d**) counties with a CSN/IMPROVE monitor. The x-axis marker denotes the mean concentration (and the error bars depict the 95th CI) in annual mean (2019) concentrations across all sites. The metals commonly associated with anthropogenic emissions are colored red, while the metals associated with natural emissions are colored green. Error bars represent 95th percentile confidence interval. Confidence intervals were estimated using 10,000 random samples with replacement (bootstrap sampling) of size n=138 and 25 (the number of highly segregated and well-integrated counties, respectively) in panel a, n=138 and 83 (the number of highly segregated and moderately counties, respectively) in panel b, and n=76 (an equal number of highly segregated, moderately segregated, and well-integrated counties) in panels c and d.

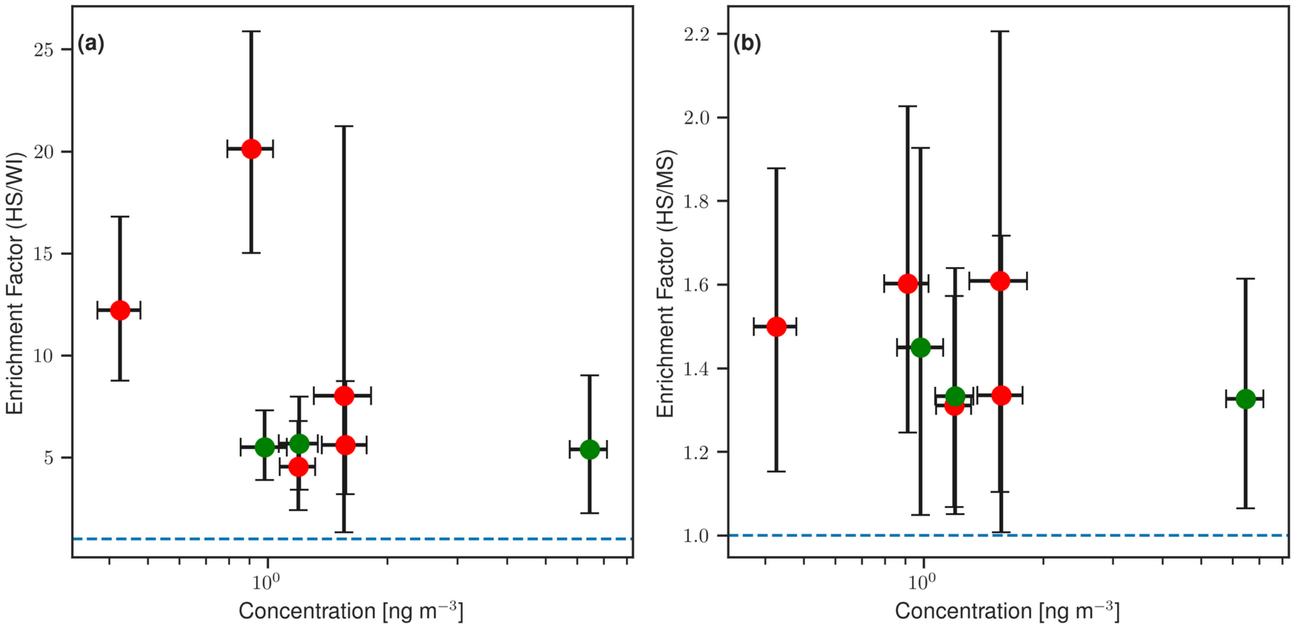

Figure S14. Sensitivity analysis on the enrichment factor (the ratio of the mean) concentration between counties characterized as highly segregated ("HS") to well integrated ("WI") and highly segregated and moderately segregated ("MS") where annual mean concentrations of trace metals below the minimum detectable limit of a station are replaced by the limit of detection divided by the square root of 2. Error bars represent 95th percentile confidence interval. Confidence intervals were estimated using 10,000 random samples with replacement (bootstrap sampling) of size n=47, 165, and 16 for highly segregated, moderately segregated, and well-integrated counties, respectively.

References

1. Morello-Frosch, R. & Jesdale, B. M. Separate and Unequal: Residential Segregation and Estimated Cancer Risks Associated with Ambient Air Toxics in U.S. Metropolitan Areas. Environ. Health Perspect. 114, 386–393 (2006).
